# Supplementary material for: MetaQTL: a package of new computational methods for the meta-analysis of QTL mapping experiments
Source: BMC Bioinformatics. 2007 Feb 8;8:49. doi: 10.1186/1471-2105-8-49 (PMC1808479; doi:10.1186/1471-2105-8-49)
Supplement: Additional File 7 — MetaQTL Package : jar file and tutorial. This Zip archive contains both the MetaQTL JAR file and the files of the tutorial. [file 1471-2105-8-49-S7.zip › org.inra.metaqtl/doc/org/thalia/bio/util/BioUtilities.html]

BioUtilities


|  |  |  |
| --- | --- | --- |
| **Overview** \| **Package** \| **Class** \| **Use** \| **Tree** \| **Deprecated** \| **Index** \| **Help** | |  |
| **PREV CLASS**   **NEXT CLASS** | **FRAMES**    **NO FRAMES**     **All Classes** |  |
| SUMMARY: NESTED \| FIELD \| CONSTR \| METHOD | DETAIL: FIELD \| CONSTR \| METHOD |  |


---


## org.thalia.bio.util Class BioUtilities

```
java.lang.Object
  org.thalia.bio.util.BioUtilities
```

---

``` public class BioUtilities extends java.lang.Object ```

General comments must be added here.

**Author:**
:   Julien Cornouiller
    Additions must be added here.

---

**Constructor Summary**

`BioUtilities()`


| **Method Summary** | |
| --- | --- |
| `static void` | `mergeGenome(IBioGenome g1, IBioGenome g2)`             This method merges two genomes in a single one : `g1` is modified and will contain the result of the merge. |
| `static IBioLocus[]` | `sortLociByPosition(IBioLocus[] loci)`             Sort tab with loci |

**Methods inherited from class java.lang.Object**

`clone, equals, finalize, getClass, hashCode, notify, notifyAll, toString, wait, wait, wait`

**Constructor Detail**

### BioUtilities

```
public BioUtilities()
```


**Method Detail**

### sortLociByPosition

```
public static IBioLocus[] sortLociByPosition(IBioLocus[] loci)
```

:   Sort tab with loci

    :   **Parameters:**: `loci` -

---


### mergeGenome

```
public static void mergeGenome(IBioGenome g1,
                               IBioGenome g2)
```

:   This method merges two genomes in a single one : `g1` is
    modified and will contain the result of the merge.


---


|  |  |  |
| --- | --- | --- |
| **Overview** \| **Package** \| **Class** \| **Use** \| **Tree** \| **Deprecated** \| **Index** \| **Help** | |  |
| **PREV CLASS**   **NEXT CLASS** | **FRAMES**    **NO FRAMES**     **All Classes** |  |
| SUMMARY: NESTED \| FIELD \| CONSTR \| METHOD | DETAIL: FIELD \| CONSTR \| METHOD |  |


---
